# A Numerical Measurement Method for Dynamic Granular Materials Based on Computer Vision

**DOI:** 10.3390/ma15103554

**Published:** 2022-05-16

**Authors:** Hao Liu, Yuxing Nie, Man Chen, Shunkai Liu, Ashiru Mohammed

**Affiliations:** 1 School of Civil Engineering, Central South University, Changsha 410075, China; sundayritian@csu.edu.cn (H.L.); mohammeda@abu.edu.ng (A.M.); 2 Department of Civil and Environmental Engineering, Hong Kong Polytechnic University, Hong Kong 999077, China; 21054468g@connect.polyu.hk; 3 School of Electrical and Information Engineering, Changsha University of Science and Technology, Changsha 410114, China; 19205060770@stu.csust.edu.cn; 4 School of Civil Engineering, Hunan University of Science and Technology, Xiangtan 411201, China; 5 Department of Civil Engineering, Ahmadu Bello University, Zaria 810107, Nigeria

**Keywords:** dynamic granular materials, numerical measurement, computer vision, video instance segmentation

## Abstract

Granular materials are widespread in nature and human production, and their macro-mechanical behavior is significantly affected by granule movement. The development of computer vision has brought some new ideas for measuring the numerical information (including the amount of translation, the rotation angle, velocity, acceleration, etc.) of dynamic granular materials. In this paper, we propose a numerical measurement method for dynamic granular materials based on computer vision. Firstly, an improved video instance segmentation (VIS) network is introduced to perform end-to-end multi-task learning, and its temporal feature fusion module and tracking head with long-sequence external memory can improve the problems of poor video data quality and high similarity in appearance of granular materials, respectively. Secondly, the numerical information can be extracted through a series of post-processing steps. Finally, the effectiveness of the measurement method is verified by comparing the numerical measurement results with the real values. The experimental results indicate that our improved VIS obtains an average precision (AP) of 76.6, the relative errors and standard deviations are maintained at a low level, and this method can effectively be used to measure the numerical information of dynamic granular materials. This study provides an intelligent proposal for the task of measuring numerical information of dynamic granular materials, which is of great significance for studying the spatial distribution, motion mode and macro-mechanical behavior of granular materials.

## 1. Introduction

Granular materials (such as coarse-grained soil) change from a loose state to a dense one, which is the result of internal mesoscopic structure changes caused by granule movement [1,2,3]. Meanwhile, translation and rotation [4], as two main forms of movement, have a significant impact on the macroscopic mechanical behavior (especially deformation) of dynamic granular materials [5]. Hence, it is necessary to measure the numerical information of dynamic granular materials (including the amount of translation, the rotation angle, velocity, acceleration, etc.). At present, the main method for obtaining and analyzing the numerical information of dynamic granular materials is the discrete element method (DEM), which was first proposed by Cundall and Strack in the 1970s [6], and has been continuously addressed and developed by many scholars [7]. However, the numerical model itself and its statistical results lack effective data verification, so it is difficult to be accepted universally. In general, calibration of numerical simulation parameters through physical experiments is one of the most effective means to ensure the reliability of models and results. However, numerical measurement of granule movement becomes an important prerequisite in physical experiments. Earlier methods only measured the movement of a small number of granules. With the development of technology, particle tracking velocimetry (PTV), particle imaging velocimetry (PIV), laser speckle velocimetry (LSV) and other technologies have emerged [8] that are able to track and measure a large number of granules, among which PTV technology is widely used in granule flow experiments [9]. However, the above methods are prone to aliasing when there are too many granules, which may result in low accuracy and a portion of the granules not being measured. Therefore, in order to improve the identification accuracy and measure more granules, so as to carry out more comprehensive and accurate analysis of granule movement, it is very necessary to propose a reliable numerical measurement method for granule movement.

In the field of computers, there is the dynamic video instance segmentation (VIS) task [10], in which the detection, segmentation, and tracking of instances in a video are performed simultaneously. Most VIS models [10,11,12,13,14] follow a two-stage paradigm. Taking MaskTrack R-CNN [10] as an example, feature maps are extracted by ResNet [15] and a feature pyramid network (FPN) [16], and then a series of candidate proposals are produced through a region proposal network (RPN) [17]. In the second stage, **features can be extracted by region of interest align (RoIAlign) and further fed into** each sub-task head, such as the box head, mask head and tracking head, to predict bounding boxes and instance masks, and perform object tracking. As one of the most challenging tasks in computer vision at present, VIS has certain application prospects for tasks that require video-level object masks, such as video editing, autonomous driving and augmented reality. We introduce VIS to perform the numerical measurement of dynamic granule materials because it can the extract mask trajectories of granule materials from videos.

Compared to static images, video frames usually offer poor image quality as a result of the acquisition equipment. Phenomena such as vibration and uneven light often exist in the environment, which may also adversely affect visual processing. Therefore, we add a temporal feature fusion module [18] to the VIS model. This module can combine the context information to improve the above problems of video frames, making the VIS model more suitable for video data. In addition, granular materials are usually densely packed and have a high similarity in appearance, which may cause object tracking errors. A single instance ID assignment error may invalidate the entire numerical chain and have a devastating impact on numerical measurement. We introduce a tracking head with long-sequence external memory to deal with this issue, so it is able to fully consider the features within multiple sequences to increase the robustness of tracking.

The above improved VIS network can detect, segment, and track granular materials frame by frame, and we also design a series of post-processing steps to measure the amount of translation, the rotation angle, velocity and acceleration of dynamic granule materials. Specifically, our work delivers the following contributions:

1. We combine computer vision and the numerical measurement task to propose a numerical measurement method for dynamic granular materials. This method is mainly based on the VIS, which is able to realize end-to-end multi-task learning and simultaneously detect, segment and track dynamic granular materials;
2. We analyze the properties of video data and granular materials to improve the VIS network. A temporal feature fusion module and tracking head with long-sequence external memory are introduced to make the VIS network more suitable for the numerical measurement of dynamic granular materials;
3. A variety of effective post-processing steps such as the extraction of centroid and long axis, ellipse fitting, and pixel-actual distance calibration are used to obtain the amount of translation, the rotation angle, velocity and acceleration of dynamic granular materials;
4. A set of experimental equipment is designed to collect dynamic granule videos and then the numerical results of dynamic granular materials are measured by the proposed method. The amount of translation, the rotation angle, and the velocity and acceleration of granular materials are compared with true results to verify the effectiveness of the proposed method.

## 2. Method

### 2.1. Method Framework

**Figure 1:**
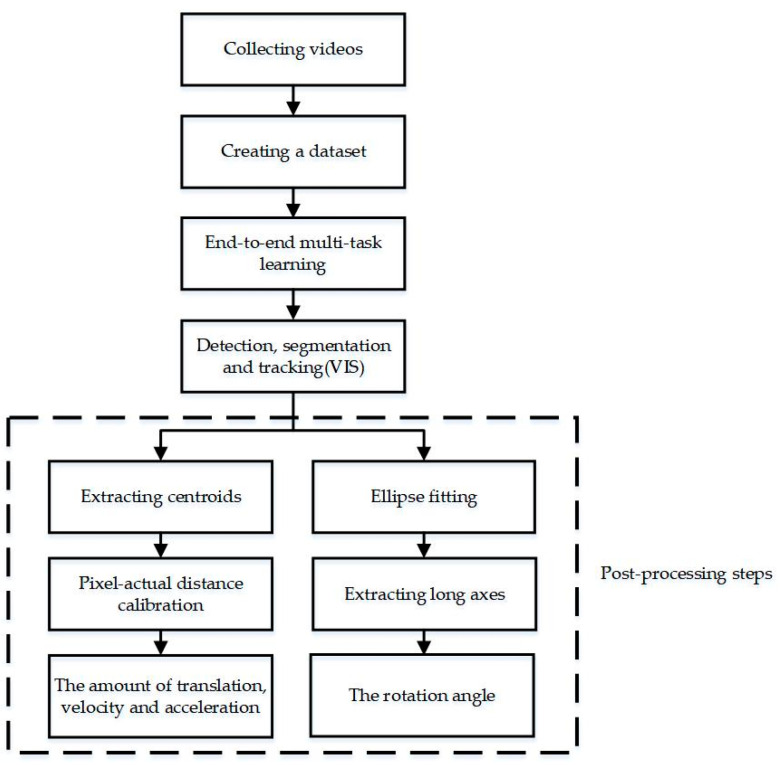
Method framework. The main processes of our proposed method are: Collecting videos, creating a dataset, end-to-end multi-task learning, VIS and post-processing steps. VIS is the key process for numerical information measurement of granular materials.

The overall method framework is illustrated in Figure 1. Firstly, videos of granular materials are collected and annotated to create a dataset. Secondly, the improved VIS network can be trained by end-to-end multi-task learning and dynamic granular materials can be detected, segmented and tracked simultaneously. Then, the centroids of granules are extracted, and ellipse fitting is performed on the masks. The amount of translation and the rotation angle are calculated by the changes of the centroids and fitted ellipse major axis angles, respectively. Further, the velocity and acceleration of dynamic granular materials could also be extracted. In addition, it is necessary to calibrate the pixel distance and actual distance when measuring translation, velocity, and acceleration of granular materials.

### 2.2. An Improved Video Instance Segmentation Network

#### 2.2.1. Overall Network Architecture

**Figure 2:**
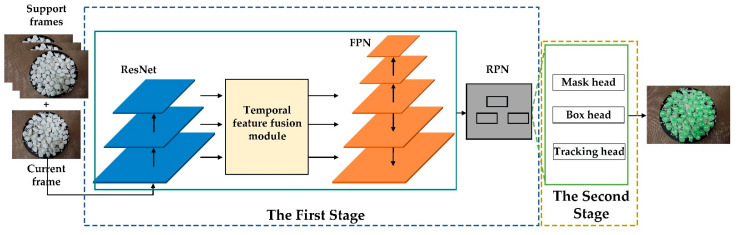
The overall architecture of our improved VIS. Our improved VIS consists of two stages and the first stage is composed of ResNet, temporal feature fusion module, FPN and RPN, where the added temporal feature fusion module can aggregate the feature information of support frames to enhance the feature response in current frame. The second stage can extract features by RoIAlign, and then the box head, mask head and tracking head can achieve bounding box regression, mask generation, and tracking, respectively. RoIAlign is omitted here.

The overall architecture of our improved VIS, which simultaneously detects, segments and tracks objects in videos through a two-stage multi-task learning approach, is shown in Figure 2. In the first stage, feature maps are produced from the input video frames by ResNet [15] and the temporal fusion module can aggregate the feature information of support frames to enhance the feature response in current frame. Then, the multi-scale feature maps are generated through FPN [16] and multiple candidate objects are extracted with RPN [17] to generate a series of candidate proposals. In the second stage, the aligned RoI features are input into the box head, mask head and tracking head. The box head and mask head are inherited from MaskTrack R-CNN [10], which can achieve bounding box regression and mask generation, respectively. In Section 2.2.2 and Section 2.2.3, we will analyze the design motivation and detailed structure of the temporal fusion module and tracking head with long-sequence external memory.

#### 2.2.2. Temporal Feature Fusion Module

The video data are often fuzzy and low in quality because of some equipment factors such as lens defocus and movement blur, so there is a large quality gap between ordinary static image data. In addition, unfavorable conditions such as vibration and uneven lighting in the collection environment for granular materials can also affect the quality of the data. In response to the above disadvantages, a temporal fusion module is added to our VIS model.

**Figure 3:**
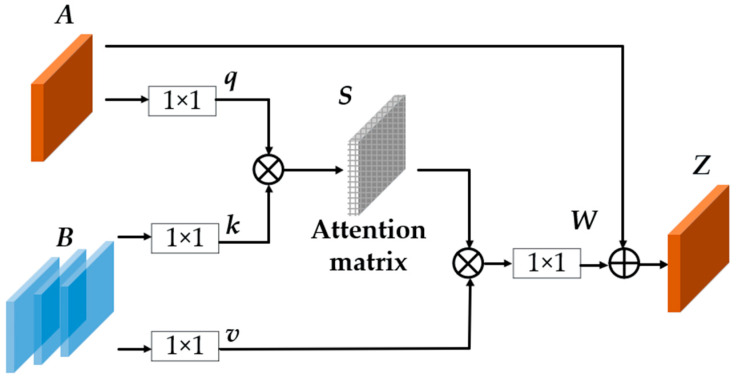
Temporal feature fusion module. This module can convert the feature map A from ResNet into the enhanced feature map Z, and Z not only preserves some informative key visual semantics of the current frame, it also incorporates useful contextual information regarding the support frames in same video.

As shown in Figure 3, this module can enrich the features of current frame by support frames. Firstly, the feature map A output from ResNet can be converted into a new feature map q by a 1×1 convolution and nonlinear activation, and this new feature map q encodes the key information (object category, object location and mask) in the current frame. Secondly, the feature map B of the support frames is encoded into k and v by two parallel 1×1 convolutions and nonlinear activations. The attention matrix S can be obtained by computing the inner product of q and k, so S is related to each position in q and k. Then the attention matrix S can be used to aggregate the feature of v to get a new feature map, and the new feature map fuses temporal information from the support frames. Finally, the new feature map can be transformed into feature map W by a 1×1 convolution and nonlinear activation, and then W is added to the original feature map A to acquire the enhanced feature map Z. The overall process can be summarized in the form of the following equations:

(1) S=k⊙q

(2) $$W=F(v⊙\frac{\exp(S\left(:,j\right))}{\sum_{i=1}^{N_{all}}\exp(S\left(:,i\right))})$$

(3) Z=W⊕A

where ⊙ is the inner product. i and j are the indices of each position in the similarity matrix and feature map, respectively. $N_{all}$ is the total number of positions. F is the transformation function which corresponds to 1×1 convolution and nonlinear activation.⊕ is summing up. The enhanced feature map Z not only preserves some informative key visual semantics of current frame, but also incorporates useful contextual information for support frames in the same video.

#### 2.2.3. Tracking Head with Long-Sequence External Memory

**Figure 4:**
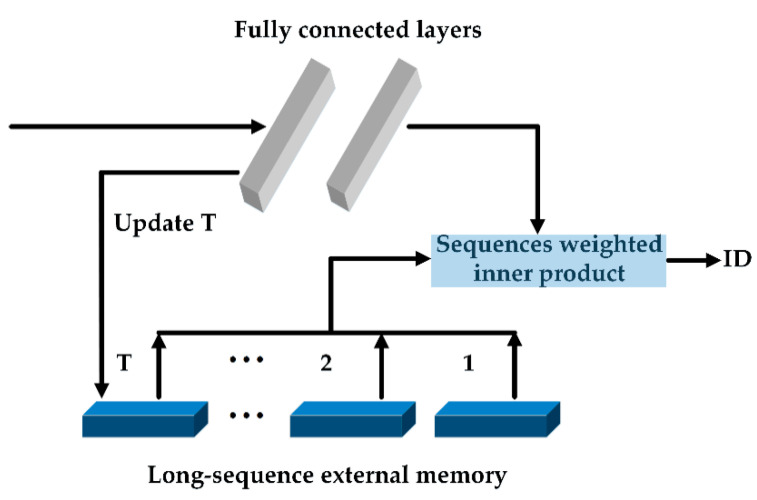
Tracking head structure. Our tracking head consists of two main parts: fully connected layers and long-sequence external memory, which can assign instance IDs to candidate objects in the current frame by calculating and comparing sequences weighted inner products.

Granular materials are often densely packed and the similarity in appearance between granular materials is high, which causes difficulties in tracking. Recently, per-clip models [19,20,21] were reported to obtain better VIS effects by aggregating multi-frame information. Inspired by these models, we design a tracking head that can comprehensively compare instance similarity across multiple frames to enhance tracking performance. The structure is shown in Figure 4, and this tracking head mainly includes two fully connected layers and a long-sequence external memory. Two fully connected layers can map features for candidate objects. The long-sequence external memory can store the features of previous instances. We use the inner products to represent the correlation between candidate object and previous instances, and each previous instance in memory can hold features of at most L sequences. Specifically, for a candidate object i, its inner product with the previous instance j already existing in the long-sequence external memory can be expressed as sequences weighted inner product:

(4) $$\phi_{ij}=\overset{L}{\underset{l=1}{\sum }}\gamma_{l}\phi_{i}^{T}\phi_{j_{l}}$$

where l is the sequence index. $\gamma_{l}$ is the sequence discount factor at l. $\phi_{i}$ is the feature of candidate object i and $\phi_{j_{l}}$ is the feature of instance j at l. $\phi_{i}^{T}\phi_{j_{l}}$ is the inner product of $\phi_{i}$ and $\phi_{j_{l}}$. *For those instances that do not have* L *sequences in memory, we only compute the inner product of existing instances for fair comparison.*

In the training phase, we use $L_{tr}$ reference frames and a query frame to train our tracking head. For reference frames, we extract features from their ground-truth instance regions and save them to the long-sequence external memory. Instances between reference frames are also matched by ground truth regions. The sequence discount factor $\gamma_{l}$ is the average of number of reference frames *because the reference frames are randomly selected from video frames during training, and* $\gamma_{l}$ *can be expressed as:*

(5) $$\gamma_{l}=\frac{1}{L_{tr}}$$

In the inference phase, we sequentially process each frame in an online fashion. Each current frame has $L_{in}$ corresponding sequences, and the sequence discount factor $\gamma_{l}$ is related to the frame sequence number of sequences in video:

(6) $$\gamma_{l}=\frac{f_{l}}{\sum_{\tilde{l}=1}^{L_{in}}f_{\tilde{l}}}$$

where $f_{l}$ is the frame sequence number of $f_{l}^{th}$ sequence in video and $f_{\tilde{l}}$ is the frame sequence number of $f_{\tilde{l}}^{th}$ sequence.

Finally, the probability of assigning instance ID x to candidate object i is calculated by Softmax, and can be expressed as:

(7) $$p_{i}\left(x\right)=\left\{\begin{array}{l} \frac{e^{\phi_{ix}}}{1+\sum_{j=1}^{N}e^{\phi_{ij}}},x\in \left[1,N\right]\\ \frac{1}{1+\sum_{j=1}^{N}e^{\phi_{ij}}},x=0 \end{array}\right.$$

where N is the number of previous instances. x=0 means that object i is a new instance and $x\in \left[1,\;N\right]$ means that object i belongs to one of the previous N instances. External memory is dynamically updated when an instance ID is assigned to a new candidate object successfully. If the candidate object belongs to an existing instance ID, we replace the feature of the farthest sequence in memory with feature of new candidate object. If the candidate object does not have a corresponding instance ID that can be assigned, the feature of candidate object is inserted into external memory and a new instance ID is created. Our tracking head can fully consider the features within L sequences in instance ID assignment, and increase the robustness of tracking for the multi-instance environment and granular materials with high feature similarity.

#### 2.2.4. Loss Function

The loss function of the VIS model consists of *four* sub-task *losses: classification, detection box regression, segmentation and tracking,* which can be expressed as:

(8) $$L=L_{cls}+L_{box}+L_{mask}+L_{track}$$

where $L_{cls}$, $L_{box}$ and $L_{mask}$ are the same losses as in Mask R-CNN [22]. $L_{track}$ is the cross-entropy loss similar to MaskTrack R-CNN [10].

### 2.3. Post-Processing Steps

To measure the amount of translation, velocity and acceleration, the centroids of granules need to be extracted first. We determine the abscissa and ordinate of the centroid independently in the x and y directions because the segmented mask is two-dimensional. Specifically, the coordinates of centroids in the x (y) direction are calculated by bisecting the number of pixels on the left and right (up and down) sides.

The centroids of granular materials can be extracted by above operation and then subtracted from the extracted values of the first frame to acquire the amount of translation. The values of velocity and acceleration in x and y directions can be obtained by taking the derivative and second derivative. It is necessary to perform pixel-actual distance calibration, because the unit for the above numerical values is pixels. We complete the calibration in a simple way, which can be expressed as follows:

(9) $$k=\frac{S_{\mathrm{act}}}{S_{\mathrm{pix}}}$$

where k is defined as the actual distance corresponding to a pixel. $S_{\mathrm{act}}$ is the actual distance and $S_{\mathrm{pix}}$ is the pixel distance.

**Figure 5:**
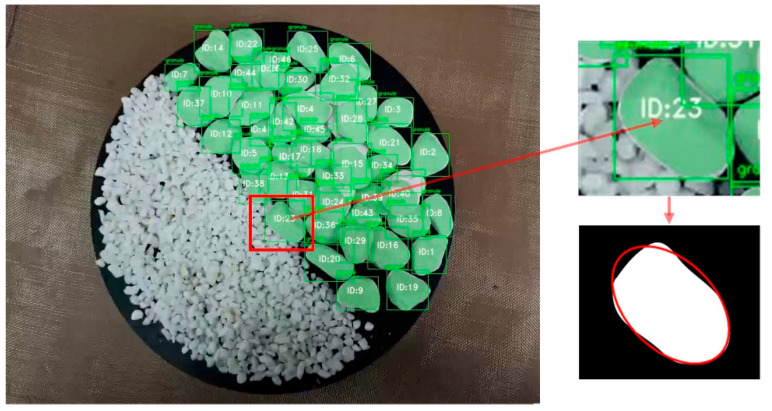
Ellipse fitting method. This method can fit masks after segmentation into ellipses and support the subsequent measurement of rotation angles.

The measurement of rotation angle is more complicated, so granular materials need to be fitted. There are many fitting methods, and the ellipse fitting method is the most suitable one for the task of movement information detection [23]. Figure 5 shows the effect of ellipse fitting. Then, the rotation angle can be successfully approximated on the basis of changes in the major axis angles of the fitted ellipses.

## 3. Experiment and Analysis

### 3.1. Experimental Equipment and Parameter

**Figure 6:**
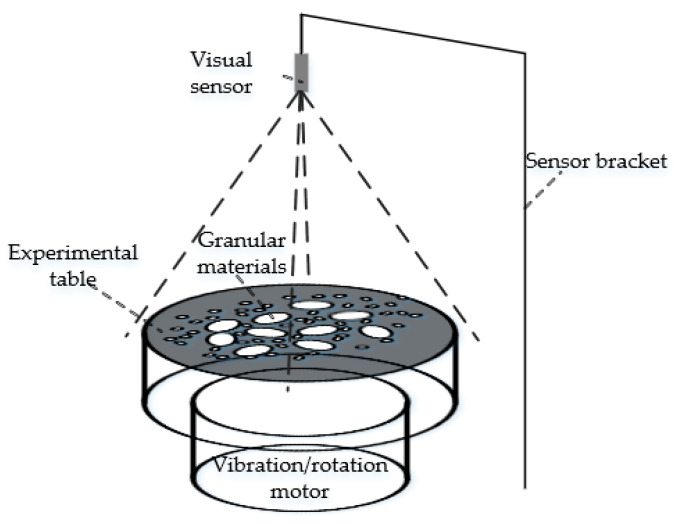
Experimental equipment. Our experimental equipment mainly consists of five components: A visual sensor for collecting videos, a sensor bracket for fixing the visual sensor, an experimental table for bearing granular materials, a motor for providing vibration or rotation load and some granular materials.

As shown in Figure 6, we designed a set of experimental equipment to monitor and record the videos of granular materials. It includes an experimental table, coarse granular materials, fine granular materials, a vision sensor and a sensor bracket. The experimental table in this study is a circular table with a diameter of 32 cm, which has with two different modes of vibration and rotation. In rotation mode, the speed can be set to 0–1.71 rad/min. The vision sensor is located 50 cm above the experimental table and is fixed by the sensor bracket. Coarse granular material and fine granular materials size range from 20 mm to 30 mm and from 2.5 mm to 7.5 mm, respectively.

The entire VIS network was trained for 12 epochs with an NVIDIA GeForce RTX3070. The backbone of our VIS network is ResNet 101 [15] with FPN [16], which are pretrained on MSCOCO dataset [24] to quicken the convergence speed. During the training phase, the model also needs to sample other frames in video to help the training of temporal feature fusion module and tracking head. For each input frame, we randomly selected five frames from the same video, and two of which were chosen as support frames according to CompFeat [18]. If a video frame belongs to both support frames and reference frames, the probability of assigning instance IDs will be affected by this frame, so three frames serve as reference frames for the tracking head and L is set to 3 during the training phase. The weights of both the pre-trained backbone network and sub-task headers were updated during training. During the inference phase, four additional frames from the test video are treated as support frames and the number of sequences is five, because testing with more information can help improve VIS performance [18]. In addition, the tracking of the evaluation process also incorporates other cues, such as semantic consistency, spatial correlation and detection confidence, as powerful post-processing techniques to improve the robustness of the tracking [10].

### 3.2. Dataset

**Figure 7:**
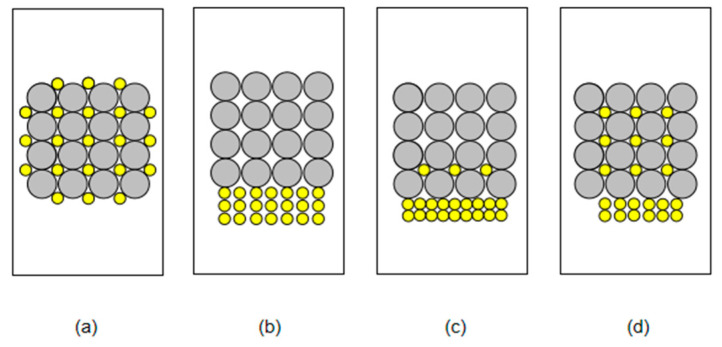
Schematic diagram of mixing degree. (**a**) The mixing degree of 100%. (**b**) The mixing degree of 0%. (**c**) The mixing degree between 0% and 50%. (**d**) The mixing degree between 50% and 100%.

We utilized the degree of mixing to express the distribution of coarse granules and fine granules, and divided the degree of mixing into four levels. Figure 7 presents different mixing degrees, with Figure 7a representing 100%, which means that the coarse granules and fine granules are uniformly mixed; meanwhile, Figure 7b presents 0% mixing degree, Figure 7c represents a degree of mixing that is between 0% and 50%, which means that a small part of the granules are mixed, and Figure 7d presents a mixing degree of between 50% and 100%, which indicates that most of the granules are mixed.

As shown in Table 1, we collected videos of dynamic granular materials with a total duration of 29,092 frames (about 970 s) using the experimental equipment. Considering that the movement amplitude of vibrating granular material is low, we selected one frame for labeling from every 60 frames of the vibrating videos. However, videos with rotating granular materials have a large amount of movement, so we marked one frame from every 30 frames of the rotating videos. The duration of each video varied from 5–45 s and the label files followed MSCOCO’s style [24]. We only performed VIS on coarse granular materials in this experiment, because the labeling of fine granular materials is too difficult. In addition, about one-third of the videos had problems such as lens defocus and uneven lighting to enhance the robustness of the network and verify the model’s adaptability to image quality problems. We marked 706 frames and all videos were randomly divided into training videos and validation videos according to the ratio of about 6:1.

### 3.3. Evaluation Indicators

We set up the evaluation indicators on the basis of two aspects: visual processing and numerical measurement. The common average precision (AP) is used to reflect the effect of visual processing. Our AP can be calculated in the same way as in the image except for the intersection-over-union (IoU). This IoU is extended from the image to the video sequence, which can represent the degree of overlap between the predicted mask sequence and the real mask sequence in the entire video sequence [10]. The numerical measurement evaluation indicators can be divided into two parts: relative error and standard deviation. The relative error is the ratio of absolute error caused by the measurement to true value, and mainly includes two parts: the relative error of translation $E_{T}$ and the relative error of rotation $E_{R}$, which reflects the confidence of the measurement results obtained using our method:

(10) $$E_{T}=\frac{1}{VMN}\overset{V}{\underset{v=1}{\sum }}\overset{M}{\underset{m=1}{\sum }}\overset{N}{\underset{n=1}{\sum }}\left|\frac{U_{m,v}^{n}-u_{m,v}^{n}}{u_{m,v}^{n}}\right|$$

(11) $$E_{R}=\frac{1}{VMN}\overset{V}{\underset{v=1}{\sum }}\overset{M}{\underset{m=1}{\sum }}\overset{N}{\underset{n=1}{\sum }}\left|\frac{W_{m,v}^{n}-w_{m,v}^{n}}{w_{m,v}^{n}}\right|$$

where V is the number of videos in the validation set. M is the number of frames and N is the number of granular materials in the frames. Therefore, VMN represents the total number of measurements performed on the validation set. $U_{m,v}^{n}$ and $W_{m,v}^{n}$ respectively refer to the amount of translation and rotation angle of the $n^{th}$ granule of $m^{th}$ frame in $v^{th}$ video, calculated by our method. $u_{m,v}^{n}$ and $w_{m,v}^{n}$ are the true amount of translation and the true rotation angle. In addition, we calculate the standard deviation of numerical measurement absolute error, which reflects the stability of our proposed measurement method. Similarly, the standard deviation can also be divided into two parts: the standard deviation of translation $\sigma_{T}$ and the standard deviation of rotation $\sigma_{R}$, which can be expressed as:

(12) $$\sigma_{T}=\sqrt{\frac{1}{VMN}\overset{V}{\underset{v=1}{\sum }}\overset{M}{\underset{m=1}{\sum }}\overset{N}{\underset{n=1}{\sum }}{\left(\left|U_{m,v}^{n}-u_{m,v}^{n}\right|-A_{T}\right)}^{2}}$$

(13) $$\sigma_{R}=\sqrt{\frac{1}{VMN}\overset{V}{\underset{v=1}{\sum }}\overset{M}{\underset{m=1}{\sum }}\overset{N}{\underset{n=1}{\sum }}{\left(\left|W_{m,v}^{n}-w_{m,v}^{n}\right|-A_{R}\right)}^{2}}$$

where $A_{T}$ and $A_{R}$ represent the average values of absolute errors of VMN measurements of translation and rotation, respectively, which can be expressed as:

(14) $$A_{T}=\frac{1}{VMN}\overset{V}{\underset{v=1}{\sum }}\overset{M}{\underset{m=1}{\sum }}\overset{N}{\underset{n=1}{\sum }}\left|U_{m,v}^{n}-u_{m,v}^{n}\right|$$

(15) $$A_{R}=\frac{1}{VMN}\overset{V}{\underset{v=1}{\sum }}\overset{M}{\underset{m=1}{\sum }}\overset{N}{\underset{n=1}{\sum }}\left|W_{m,v}^{n}-w_{m,v}^{n}\right|$$

### 3.4. Visual Processing Experiment

**Figure 8:**
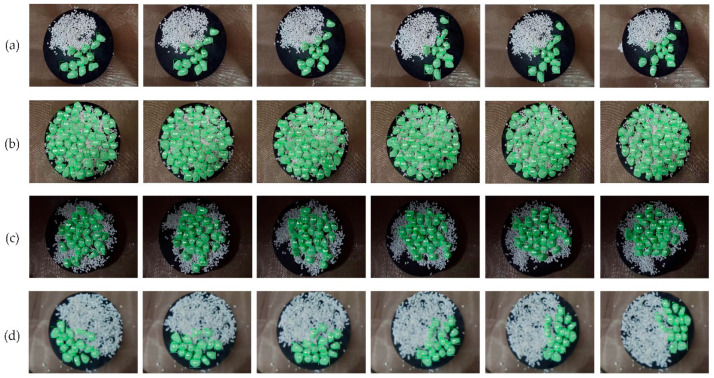
Qualitative results of visual processing experiment. (**a**) Vibrational state video with a degree of mixing of 0–50; (**b**) rotating state video with a degree of mixing of 100; (**c**) video with a degree of mixing of 100 under insufficient lighting; (**d**) video of lens defocus with a degree of mixing of 50–100. The above videos are displayed evenly at intervals of 90 frames.

We designed a series of visual processing experiments to demonstrate the effectiveness of the improve VIS network. Firstly, the evaluation indexes of visual processing were calculated to verify effect of granular materials VIS and then compared with some methods on a self-created dataset, as presented in Table 2. Secondly, we conducted ablation experiments to investigate the temporal feature fusion module and tracking head with a long-sequence external memory. Finally, qualitative experimental results on different videos are presented in Figure 8.

As shown in Table 2, our method achieves the best results in visual processing metrics. All baselines follow the idea of “tracking-by-detection”, but IoUTracker+ and Deep SORT are not trained end-to-end. These methods use an instance segmentation algorithm to segment out the mask independently on each frame and then link instances across frames by means of an object tracking algorithm. To compete fairly with end-to-end methods, the instance segmentation part of IoUTracker+ and Deep SORT was Mask R-CNN. Obviously, the overall performance of end-to-end methods is better than that of non-end-to-end methods. This is because the end-to-end approach can integrate detection, segmentation and tracking tasks in one VIS framework and optimize them jointly. In addition, the AP of our method is 2.1% higher than MaskTrack R-CNN, which shows that the temporal feature fusion module and new tracking head can bring advantages to VIS of granular materials.

As shown in Table 3, we designed a series of ablation experiments to verify the impact of each component for visual processing results. The temporal feature fusion module has a greater impact on visual processing, because the module can make full use of the contextual information of other video frames. It is worth noting that the tracking head with long-sequence external memory also has a certain improvement effect on visual processing. This is because the IoU in VIS is extended from static images to videos, and it associates the tracking effect with the AP. In summary, adding a temporal feature fusion module and improving the tracking head can achieve better visual processing results of granular materials.

Figure 8 shows the qualitative results of granular material VIS. We selected one image from every 90 frames for all videos for display and annotated the instance ID of objects inside the bounding box. Most granules in the videos can be segmented and tracked in instance dimension. The segmented masks can overlay objects well, and most granules do not have evident under-segmentation and over-segmentation. We also show the video processing results of uneven illumination and lens defocus in validation. It can be seen that our VIS model can also achieve good instance segmentation and tracking for these two adversely affected videos. The VIS of the above granular materials can obtain complete mask chains and the numerical information of granular materials can be obtained by further post-processing.

### 3.5. Numerical Measurement Experiment

As shown in Table 4, we measured the numerical information of granular materials in the validation set and calculated the measurement errors to verify the effectiveness of our proposed numerical measurement method. Calculations of measurement errors need to firstly extract true numerical results of granular materials. For granular materials in the vibrating state, a method by marking the long axes of primordial granules was developed in order to collect the true movement information. One frame per 5 s of video was selected, and the LabelMe data labeling tool was utilized to artificially mark the long axes of granules. Then, the long axis coordinates were obtained from the corresponding .json file. The amount of translation of granules can be obtained by the change of center locations of the manually marked long axis coordinates, and rotation angles can be approximated on the basis of the rotation angles of long axes. Finally, the movement information extracted with the artificial method was regarded as the true values. For rotating granular materials, we directly calculate their true amount of translation and true rotation angle results through experimental equipment.

During the experiment, we found that for a small number of granular materials, mask trajectory interruptions occur, or they are associated with other IDs because of the detection or tracking errors, which may make the entire data chain invalid. The translation and rotation errors of such granules are often huge, so we avoid these granular materials when calculating the measurement error and only count the measurement errors of the effective data chains. Effective data chains can be selected by setting a monitoring threshold for each frame of displacement, and the threshold is the average of the diameters of minor axes of all fitted ellipses. When the displacement exceeds this value, the granule is considered to have an ID assignment error, and the data chain is discarded.

It can be seen from Table 4 that the relative errors of translation and rotation can be kept at a low level, which shows the effectiveness of the proposed method. The relative errors in vibrating-type videos are large because the real values of these videos are obtained by manual calibration. The relative errors of the rotation angles of vibrating-type videos are the largest among all errors, with a value of 16.43%. This is because the rotation angles of granular materials are calculated by fitted ellipses and long axes, which need more approximation. On the other hand, the standard deviations are also maintained at a low level, whether the video is of vibrating or rotating type, which reflects the stability of our proposed measurement method. In addition, the standard deviation of vibrating videos is greater than that of rotating videos, it also because the true values of translation and rotation angles of vibrating videos are obtained by manual marking. In general, our improved VIS network and a series of post-processing steps can accurately measure the amount of translation and rotation angle of dynamic granular materials and maintain a high numerical measurement stability.

**Figure 9:**
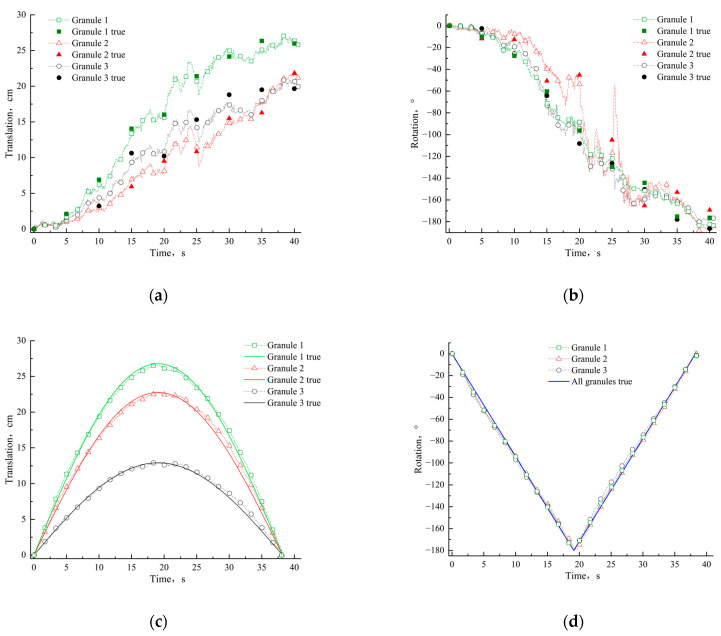
Translation and rotation curves for granular materials. (**a**,**b**) Translation and rotation of three granules in a vibrating video, where the solid points are true results by manually marking. (**c**,**d**) Translation and rotation of three granules in a rotating video, where the solid lines are the true values from the structured environment.

**Figure 10:**
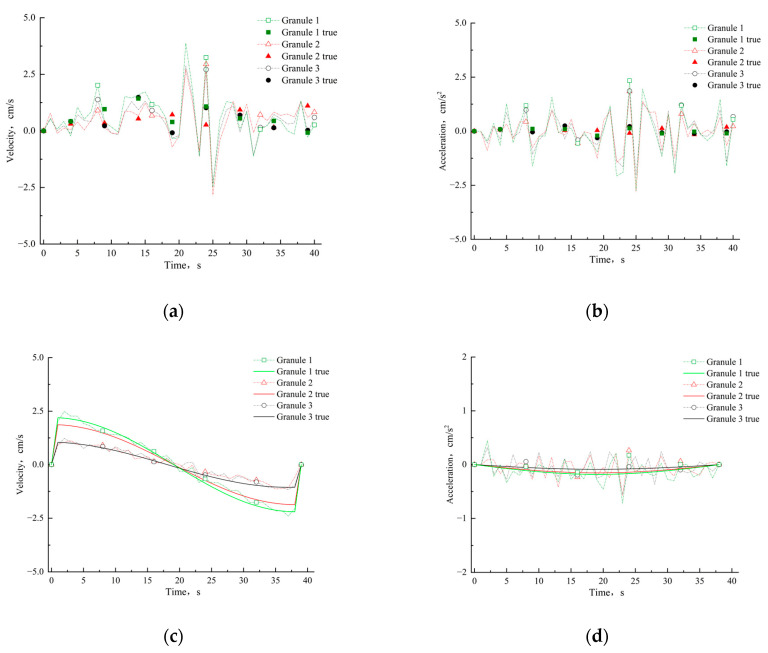
Velocity and acceleration curves for granular materials. (**a**,**b**) Velocities and accelerations of three granules in Figure 9a,b, where the solid points are the true values. (**c**,**d**) Velocities and accelerations of three granules in Figure 9c,d, where the solid lines are the true values.

As shown in Figure 9, we plot the amount of translation curves and rotation angle curves of the granules as a function of time and compare them with the true values. Considering that velocity and acceleration also have a large effect on the macroscopic mechanical behavior of dynamic granular materials, we also plot these curves (Figure 10).

In terms of general laws, the translation and rotation of granular materials in the vibrating-type video are irregular, while the translation of granular materials in Figure 9c is a trigonometric function with the same period, because these granular materials rotate at a constant speed around the center of experimental table. The rotation angle in Figure 9d is a linear function, which is also because these granular materials rotate uniformly around the center of the experimental table. The motion laws of the above granular materials are in line with expectations. It can be observed that the curves and the real scatter points are basically consistent in Figure 9a,b, and it can also be seen from Figure 9c,d that the measured curves and corresponding true curves are generally consistent, which shows that our proposed numerical measurement method can accurately measure the translation and rotation of granular materials in two types of vibration and rotation. 

It is worth noting that the trend of the amount of translation curve of granule 2 in Figure 9a is generally consistent with the trend of curves of other granules under the same vibrational load, but the rotation angle curve of granule 2 in Figure 9b shows some differences compared to the rotation angle curves of granule 1 and granule 3. To explain the reason for the occurrence of the above phenomenon, we searched for granule 2 in the corresponding video and found its shape to be close to that of a standard circle. In our proposed method, the rotation angle of granular material is calculated by fitting the mask after segmentation into an ellipse and then using the long-axis rotation angle to approximate the rotation angle of granular material. Since the shape of granule 2 is close to that of a standard circle, the above-mentioned method may generate a certain error in measuring rotation angle, resulting in the phenomenon that the rotation curve of the granule 2 in Figure 9b does not match that of granule 1 and granule 3.

Figure 10a,b reflect the velocity and acceleration of granular materials in vibrating state video and the true values are calculated from manual measurements of translation and rotation. Figure 10c,d show the velocity and acceleration of the rotating granule materials and true results calculated from the parameters of the experimental equipment. It can be seen that velocity errors and acceleration errors are maintained at a low level, which demonstrates the effectiveness of our method in measuring the velocity and acceleration of granular materials. It is worth noting that the range of the ordinate in Figure 10d is small, which causes the curve trend of the measured results and the true values to be inconsistent.

## 4. Conclusions and Outlook

In this study, a numerical measurement method for dynamic granular materials based on an improved video instance segmentation (VIS) network is proposed. Firstly, the improved VIS network can realize multi-task learning based on data annotations and simultaneously detect, segment, and track dynamic granular materials. Secondly, the adverse effects of lens defocus, uneven light, and high appearance similarity between different granular materials can be effectively dealt with by the temporal feature fusion module and new tracking head with long sequence memory. Finally, the numerical measurement of the amount of translation, the rotation angle, velocity, and acceleration of dynamic granular materials can be achieved through post-processing steps including centroid extraction, long axis extraction, ellipse fitting and pixel-actual distance calibration. The experimental results show that the improved VIS can achieve an average accuracy (AP) of 76.6. The measurement errors of translation and rotation angle are 8.95% and 16.43%, respectively, in vibrating videos, and 5.67% and 9.51%, respectively, in rotating videos with granular materials. **Standard deviations of absolute errors of translation and rotation are maintained at a low level, demonstrating the stability of our numerical measurement method.**

The method in this study can be used to accurately measure the translation, rotation, velocity and acceleration information of dynamic granular materials, and has great advantages and good application prospects in the calibration of discrete element method. It is believed that this study is of great significance to study the **spatial distribution, motion mode and macro-mechanical behavior of** granular materials. However, it is worth pointing out that the method in this paper has some shortcomings. Firstly, it is difficult to measure the numerical information of occluded granular materials, because our method relies on a visual sensor to capture videos. Secondly, our method approximates the motion space of granular materials as a two-dimensional plane in the process of extracting the numerical information of granular materials. Thirdly, this method approximately measures the rotation angles of granular materials by fitting ellipse and extracting the rotation angle of long axis, which is challenging to apply to granular materials that are close to standard circles. Finally, similar to the common risk of deep neural networks, the VIS part of our method struggles to provide a detailed theoretical derivation process. Therefore, our approach has poor interpretability compared to traditional mathematical models.

The shortcomings of the method proposed in this study will be further investigated. Firstly, we will implement the numerical measurement of obscured granular materials by obscured object detection methods in computer vision. Secondly, depth information in the experimental environment will be extracted using a depth camera, and we will combine depth information to extend granular materials from the two-dimensional plane into three-dimensional space for study. Thirdly, to address the difficulty of measuring the rotation angles of granular materials with shapes close to standard circles, we will further extract finer texture information to obtain a more accurate representations of angles. Finally, the important metric of measurement speed is not considered in this study. We will complete the VIS task with more lightweight neural network model and meet the requirements for real-time performance in real-world measurements. We also hope to strengthen the study of interpretable part in future research.

## Figures and Tables

**Table 1:** Dataset statistics.

Video Type	Degree of Mixing	0%	0–50%	50–100%	100%	Total
Vibrating	Number of videos	8	9	11	9	37
	Number of frames	3072	3755	4733	3987	15,547
	Number of marked frames	51	62	78	66	257
Rotating	Number of videos	9	8	9	8	34
	Number of frames	2930	3058	3855	3702	13,545
	Number of marked frames	97	101	128	123	449

**Table 2:** Comparison of mask AP of granular materials.

Method	AP	AP50	AP75
IoUTracker+ [25]	66.4	75.4	67.5
Deep SORT [26]	69.7	78.0	70.6
MaskTrack R-CNN [10]	74.5	85.2	75.8
Ours	76.6	88.3	78.1

**Table 3:** Comparison of ablation experiment results. “TF” refers to the temporal feature fusion module and “LM” refers to the tracking head with long-sequence external memory.

TF	LM	AP	AP50	AP75
		74.5	85.2	75.8
√		76.3 (+1.8)	87.7 (+2.5)	77.6 (+1.8)
	√	75.1 (+0.6)	86.3 (+1.1)	76.7 (+0.9)
√	√	76.6 (+2.1)	88.3 (+3.1)	78.1 (+2.3)

“√” means adding corresponding components to the VIS network.

**Table 4:** Measurement errors of the effective data chain.

Video Type	$E_{T}/\%$	$E_{R}/\%$	$\sigma_{T}/\mathrm{cm}$	$\sigma_{R}/{}^{\circ}$
Vibrating	8.95	16.43	0.47	3.41
Rotating	5.67	9.51	0.26	1.92

## Data Availability

All the research data used in this manuscript will be available whenever requested.

## Abbreviations

AP	Average precision at IoU = 0.50: 0.05: 0.95
AP50	Average precision at IoU = 0.50
AP75	Average precision at IoU = 0.75
CompFeat	Comprehensive feature aggregation approach
Deep SORT	Deep simple online and real-time tracking
DEM	Discrete element method
FPN	Feature pyramid network
ID	Identity document
IoU	Intersection-over-union
IoUTracker	Intersection-over-union tracker
LM	Tracking head with long-sequence external memory
LSV	Laser speckle velocimetry
Mask R-CNN	Mask region-based convolutional neural network
MaskTrack R-CNN	Mask track region-based convolutional neural network
MSCOCO	Microsoft common objects in context
PIV	Particle imaging velocimetry
PTV	Particle tracking velocimetry
ResNet	Residual Network
RoIAlign	Region of interest align
RPN	Region proposal network
TF	Temporal feature fusion module
VIS	Video instance segmentation
